# Supplementary material for: Novel Potential Markers of Myofibroblast Differentiation Revealed by Single-Cell RNA Sequencing Analysis of Mesenchymal Stromal Cells in Profibrotic and Adipogenic Conditions
Source: Biomedicines. 2023 Mar 10;11(3):840. doi: 10.3390/biomedicines11030840 (PMC10045579; doi:10.3390/biomedicines11030840)
Supplement: Supplementary file 1 [file biomedicines-11-00840-s001.zip › Supple Figures.pdf]

Figure S1. GO analysis for biological processes. A - network built for genes induced during myofibroblast differentiation, but inhibited during adipogenic differentiation, B - network built for genes induced during adipogenic differentiation, but inhibited during myofibroblast differentiation.

Figure S2. Immunocytochemical evaluation of NTM (green) in control (Control) MSCs and after incubation in profibrotic (F) or adipogenic conditions (Ad). Fluorescent microscopy, blue staining—nuclei stained with DAPI, scale bar is 50 mkm.
