# Supplementary figures and images for: Novel Potential Markers of Myofibroblast Differentiation Revealed by Single-Cell RNA Sequencing Analysis of Mesenchymal Stromal Cells in Profibrotic and Adipogenic Conditions
Source: Biomedicines. 2023 Mar 10;11(3):840. doi: 10.3390/biomedicines11030840 (PMC10045579; doi:10.3390/biomedicines11030840)

**A**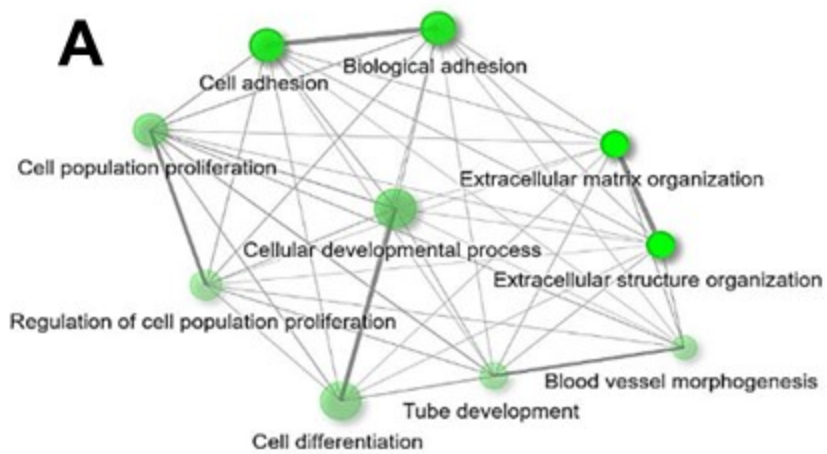**B**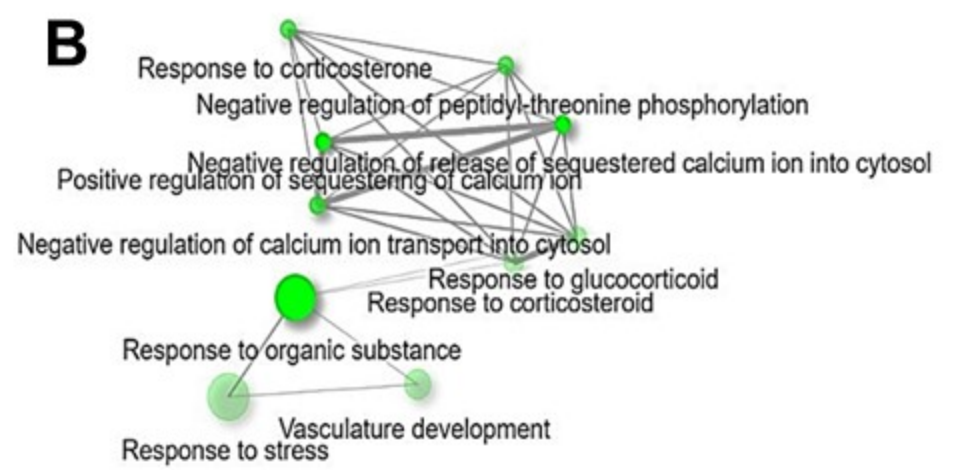

Supplement: Supplementary file 1 [file biomedicines-11-00840-s001.zip › FigS1.pdf]

Control

F

Ad

MSC#1

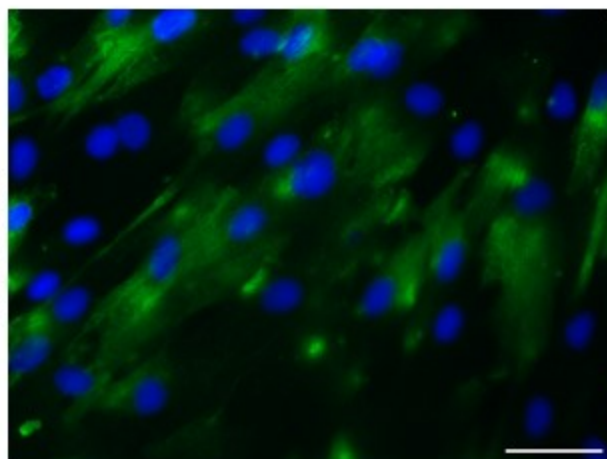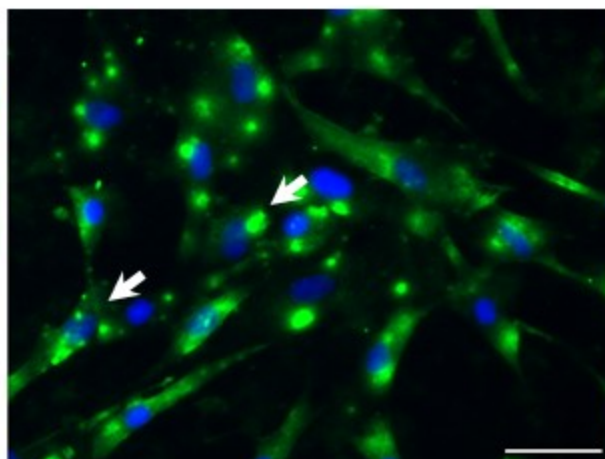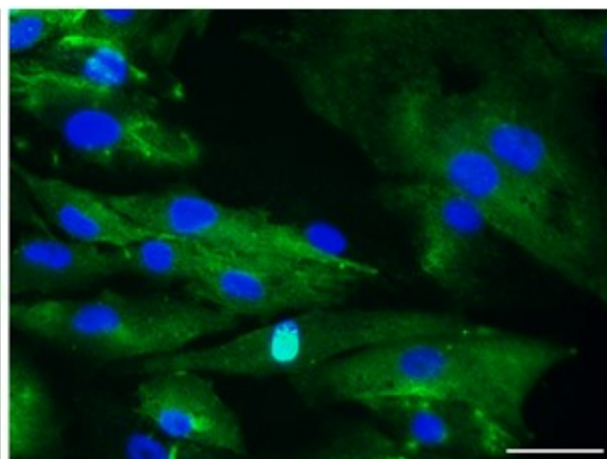

MSC#2

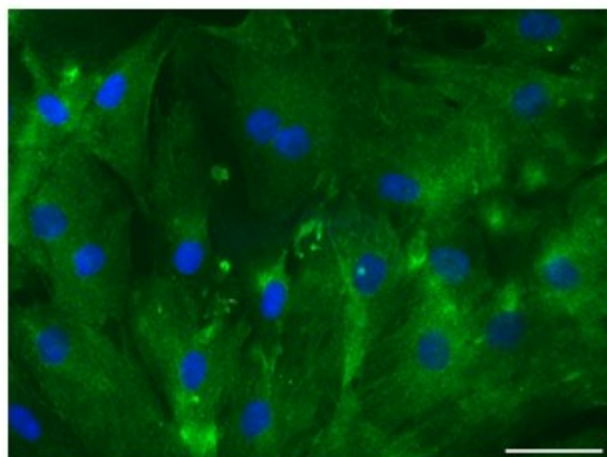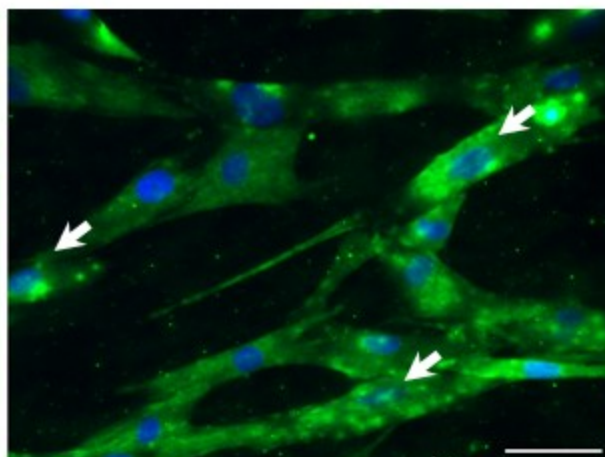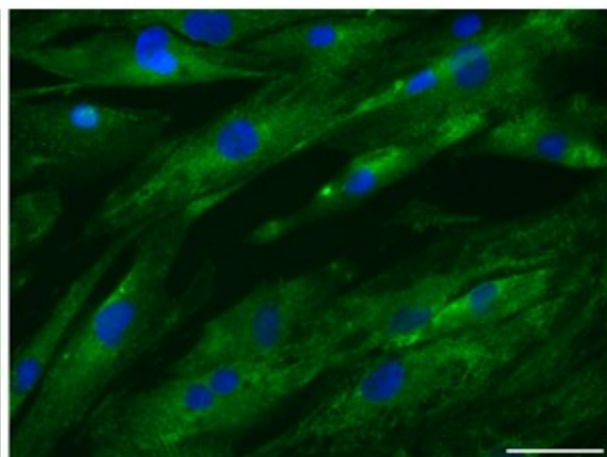

MSC#3

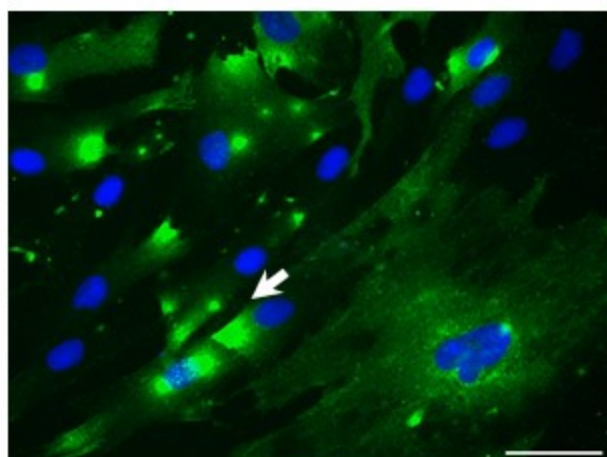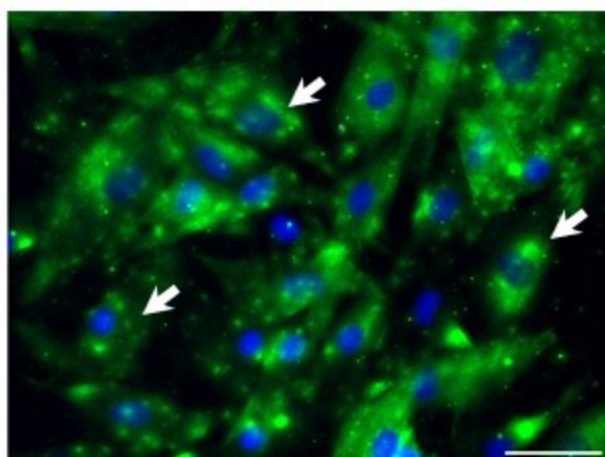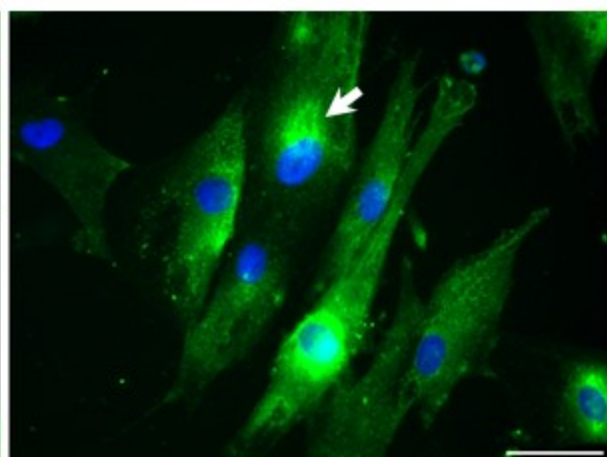

Supplement: Supplementary file 1 [file biomedicines-11-00840-s001.zip › FigS2.pdf]
